# Supplementary material for: Active fixation as an efficient coding strategy for neuromorphic vision
Source: Sci Rep. 2023 May 8;13:7445. doi: 10.1038/s41598-023-34508-x (PMC10167324; doi:10.1038/s41598-023-34508-x)
Supplement: Supplementary file 1 — Supplementary Figures. [file 41598_2023_34508_MOESM1_ESM.pdf]

Supplementary Information

Active Fixation as an Efficient Coding Strategy for Neuromorphic Vision

Simone Testa, Silvio P. Sabatini[+], Andrea Canessa[+]
University of Genoa, DIBRIS, Genoa, 16145, Italy
+ these authors contributed equally to this work

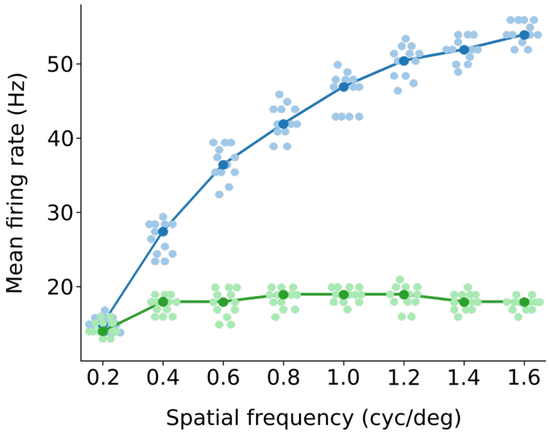

Figure S1: Comparison of the mean firing rate evoked from sinusoidal synthetic stimuli at different spatial frequencies in a range $[0.2, 1.6]$ cyc/deg in a condition where contrast is kept constant across frequency (blue line) or it is varied according to a power-law behavior of natural images (green line). Dark color dots represent the average across the 12 tested orientations of grating stimuli, single data points are displayed in light colors. We applied an horizontal jitter to single data points for the sake of visualization. Blue curve shows an amplification effect from the system, while the green one shows an equalization (whitening) of its response to varying spatial frequencies. This whitening effect is attributable to the opposing trend of natural image distribution across spatial frequencies with respect to the amplification introduced by FEMs. An amplification of system's firing activity at increasing stimulus' spatial frequency is visible when grating's contrast is kept constant (blue curve). On the contrary, a whitening effect (response equalization with spatial frequency) is shown if contrast is adjusted with spatial frequency according to natural image statistics (green).

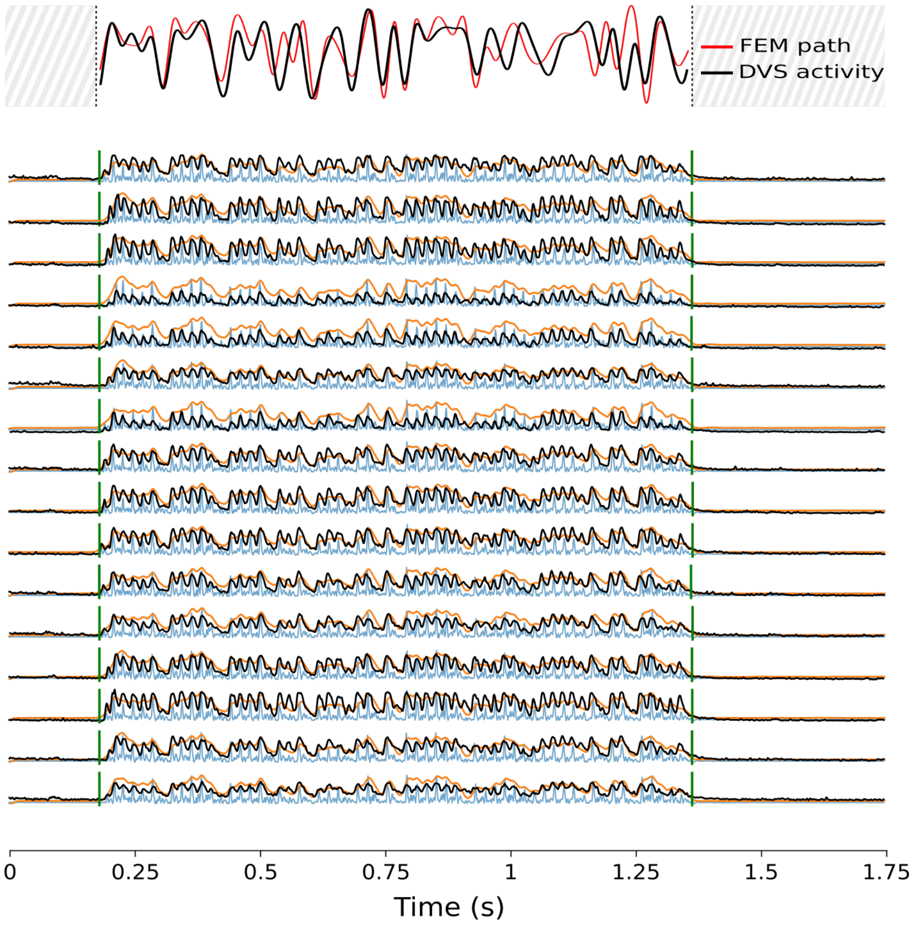

Figure S2: Examples of FEM sensor activity and IMU angular speed recordings. (Top) the distance covered by the walker during a specific FEM sequence (specific seed) is shown in red (interpolation over 60 FEM steps); the (smoothed) instantaneous firing rate of the DVS (averaged across 16 different recordings) is superimposed in black, sampled at 60 steps and interpolated. (Bottom) The 16 plots show both IMU angular speed (raw signal in light blue, smoothed signal in orange) and DVS firing activity (black). All recordings are relative to the same FEM sequence but different visual stimuli. All signals have 1 kHz sampling frequency.
